# Supplementary material for: The Fate of Transplanted Periodontal Ligament Stem Cells in Surgically Created Periodontal Defects in Rats
Source: Int J Mol Sci. 2019 Jan 7;20(1):192. doi: 10.3390/ijms20010192 (PMC6337301; doi:10.3390/ijms20010192)

Supplementary Figure

Localization of PKH26-labeled PDLSC in periodontal tissues of three more rats. Fluorescence microscopic images of periodontal tissues obtained from three more rats 4 weeks after transplantation. Lower magnification image of the section (upper). Close-up image of the red box area in upper panel is shown in lower panel. The yellow and white dotted line denotes the bottom of created periodontal defect and bone surface, respectively. PHK26-positive cells were observed in limited areas in periodontal tissues, as indicated by the yellow arrows, at the outer surface of bone (#2 and #3 rat). No PKH26-labeled cells were observed in #4 rat. Yellow arrow: PKH26 positive cells, B: bone.

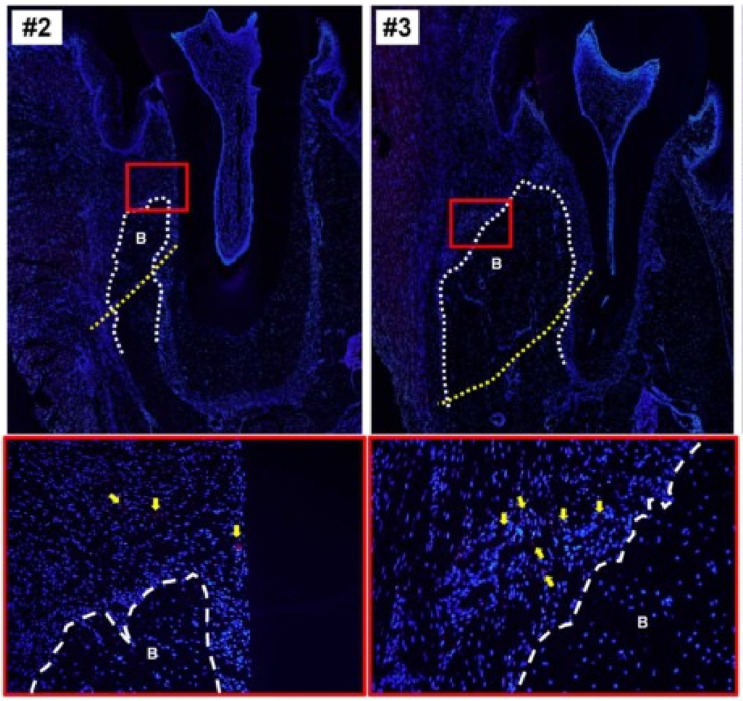

Supplement: Supplementary file 1 [file ijms-20-00192-s001.pdf]
